# NutriWomen, Novel Evidence-Based Web Platform to Support Women’s Health, Nutrition Decisions and Address Nutrition Misinformation on Social Media: Protocol for a Digital Tool Development

**DOI:** 10.3390/nu18010020

**Published:** 2025-12-19

**Authors:** Mireia Bosch Pujadas, Andreu Prados-Bo, Alessandra Wagner, Bradley C. Johnston, Andreu Farran-Codina, Montserrat Rabassa

**Affiliations:** 1 Department of Nutrition, Food Sciences and Gastronomy, Nutrition and Food Safety Research Institute (INSA-UB), Faculty of Pharmacy and Food Sciences, University of Barcelona (UB), 08028 Barcelona, Spain; mireiabosch@ub.edu (M.B.P.); afarran@ub.edu (A.F.-C.); 2 Blanquerna School of Health Sciences, Universitat Ramon Llull, 08025 Barcelona, Spain; andreupb@blanquerna.url.edu; 3 Department of Nutrition, Food Sciences and Gastronomy, Faculty of Pharmacy and Food Sciences, University of Barcelona (UB), 08028 Barcelona, Spain; 4 Department of Nutrition, College of Agriculture and Life Sciences, Texas A&M University, College Station, TX 77840, USA; bradley.johnston@tamu.edu; 5 CIBER of Frailty and Healthy Aging (CIBERFES), Instituto de Salud Carlos III, 28029 Madrid, Spain

**Keywords:** evidence-based nutrition, women’s health, health literacy, health services research, public health, media content analysis

## Abstract

**Background**: Social media, especially Instagram, spreads nutrition-related information that often lacks scientific rigor. Many women report feeling inadequately informed about women’s health by healthcare professionals, turning to social media, increasing exposure to misinformation. **Objectives**: The NutriWomen platform aims to assess the quality, methodological soundness, and credibility of nutritional health claims and dietary recommendations on Instagram targeting women across different life stages. Its goal is to develop a systematic and scientifically grounded evaluation framework to assess Instagram nutrition-related claims and the methodological quality and interpretability of their supporting evidence, and to translate the results into accessible outputs that help women make informed nutrition decisions across life stages. **Methods**: This study follows a five-stage design Stage 1 involves a retrospective content analysis of Instagram posts containing nutrition-related claims targeted at women, identified through the “Top posts” function and screened using predefined criteria. Stage 2 assesses information quality using a validated 14-item tool. Stage 3 evaluates the scientific accuracy of claims by formulating PI(E)CO(TS) questions, selecting key outcomes, retrieving evidence from PubMed and the Cochrane Database, and appraising systematic reviews with a modified AMSTAR-2 tool incorporating GRADE ratings, when available. Stage 4 develops the NutriWomen website platform to translate assessments into accessible visual summaries. Stage 5 conducts a mixed-methods study with peri-, meno-, and postmenopausal women to explore information needs and evaluate platform usability through focus groups. **Conclusions**: The NutriWomen platform will be the first website to systematically publish the results of evaluations assessing the scientific quality of nutritional health claims on Instagram targeted at women across different life stages. It will provide a replicable methodology, and a digital tool designed to empower women with trustworthy nutrition information, with the potential to enhance health literacy and promote better health outcomes.

## 1. Introduction

The lay public is increasingly interested in accessible and evidence-based sources of nutrition information. Television, search-engine-located websites and social media are the top three preferred sources to be informed about food and nutrition in Spain and Catalonia [1]. Despite the existence of European legislation, such as Regulation 1924/2006, governing nutrition and health claims, a substantial body of evidence shows that scientifically inaccurate nutrition information is widespread across digital platforms, making it challenging for consumers to trust the authenticity of the nutritional messages and claims being promoted [2,3,4].

Epidemiological data indicate that women are more likely than men to seek health and nutrition information online, yet report greater difficulty distinguishing scientific from non-scientific dietary information, particularly when judging the reliability of online nutrition content [1,5,6]. At the same time, women of reproductive age, perimenopausal women and older women express a strong interest in accessible, targeted online resources related to health and nutrition [7,8,9]. This combination of strong interest and difficulties in evaluating content underscores particular need for targeted, trustworthy resources for women.

Visual, highly interactive platforms such as Instagram, TikTok and YouTube have become major channels for disseminating misleading nutrition-related content [10]. Instagram, owned by META Platforms, Inc., is a photo- and video-sharing platform with more than 1 billion active users worldwide; its hashtag system allows users to categorize posts and search for topic-related content [11]. After Facebook, Instagram is the preferred social media platform for women seeking information on food, fitness, and health information, and hashtags such as #fitness and #food rank among the top 25 most popular on the platform [1,12]. However, Instagram has been identified as the most prevalent platform for spreading nutrition-related misinformation [13]. Women are more likely than men to engage with social media content, which increases their exposure to nutrition-related messages. Importantly, research shows that such exposure can significantly influence attitudes and behaviors towards nutrition, often in ways that are unhealthy or misinformed [14,15,16].

Common themes promoted on Instagram targeting women include reproductive health, breastfeeding, breast cancer, maternal health, and conditions such as menopause, endometriosis, or polycystic ovary syndrome; however, many posts offer recommendations that are not aligned with established clinical guidelines [13,17,18]. Social media is, therefore, a double-edged sword: while many qualified health professionals contribute to public health education, numerous self-proclaimed experts disseminate inaccurate and potentially harmful information [19]. Consequently, the influence of Instagram on nutrition-related knowledge and behavior should not be underestimated.

Despite initiatives such as Nutrimedia [4] and iHealthFacts, which rigorously appraise health and nutrition claims using systematic approaches, none specifically evaluate Instagram content targeting women’s health and nutrition needs. To the best of our knowledge, there is currently no online resource that systematically evaluates the truthfulness, information quality, and scientific confidence of claims or recommendations that are made on Instagram and correctly communicates information about nutrition and women’s health across the different life stages [20]. This research gap creates an opportunity to develop reliable, evidence-based resources specifically designed to support women’s nutrition decisions at key life stages, including the reproductive years, the menopausal transition, and older age, when nutritional and health needs change substantially [13,21,22].

The application of systematic protocols for evaluating nutritional claims on social networks aimed at women faces significant limitations in the current evidence. Addressing this critical gap, NutriWomen is an interactive, translational website designed to methodologically evaluate Instagram nutrition claims using a rigorous evidence-based evaluation framework. NutriWomen extends this approach by using a modified version of the Measurement Tool to Assess Systematic Reviews, version 2 (AMSTAR 2), to evaluate the methodological quality of systematic reviews, while incorporating GRADE ratings included within this modified tool to capture the certainty of evidence underlying nutrition-related health claims. This combined evaluation distinguishes robust evidence from findings at high risk of bias [23]. In addition, our proposed platform complements this rigorous evaluative framework with accessible and engaging formats (e.g., Healthline.com, Quackwatch.org, Nutrition.gov).

Unlike these wider platforms, NutriWomen is designed not merely as an expert-led online resource, but as a novel methodology that aims to apply a structured evaluation system both the quality of information in nutrition-related health claims on Instagram and the methodological quality of the supporting evidence in a manner that can be replicated across other health domains. Moreover, most existing studies evaluating health-related Instagram content rely on predominantly descriptive approaches, privileging content characterization over systematic appraisal of the underlying scientific evidence [24]. Among the few studies that do apply structured evaluation methods, such as the retrospective analysis of health-related claims made by naturopathic influencers on social media, none specifically target women or address the unique nutrition-related challenges they face across the life course [25].

In terms of gender perspective, although other digital platforms provide evidence-based information on women’s health (e.g., Henpicked Menopause Hub, Women Living Better), NutriWomen differs in that it focuses, as mentioned, specifically on nutrition-related health claims across women’s life stages and evaluates Instagram content using the systematic methodology outlined above. Key definitions are depicted in Table 1.

Therefore, the objectives of this project are:

(1) To identify, collect, select, and classify nutrition-related claims, as well as dietary or nutrient recommendations, disseminated on Instagram and targeted to women at their reproductive age or later aging.
(2) To assess the information quality of these claims and/or recommendations disseminated on Instagram using structured checklists of criteria.
(3) To assess the methodological soundness and scientific confidence of the supporting evidence, using the GRADE framework when available.
(4) To develop a web- and social media–based resource that translates research findings into accessible, engaging, and user-friendly formats, aimed at empowering women to make informed, evidence-based decisions to improve their nutrition-related health outcomes.
(5) To assess women’s perceptions of this resource, its usefulness, accessibility, and relevance, through a pilot study involving focus groups.

## 2. Experimental Design

**Figure 1:**
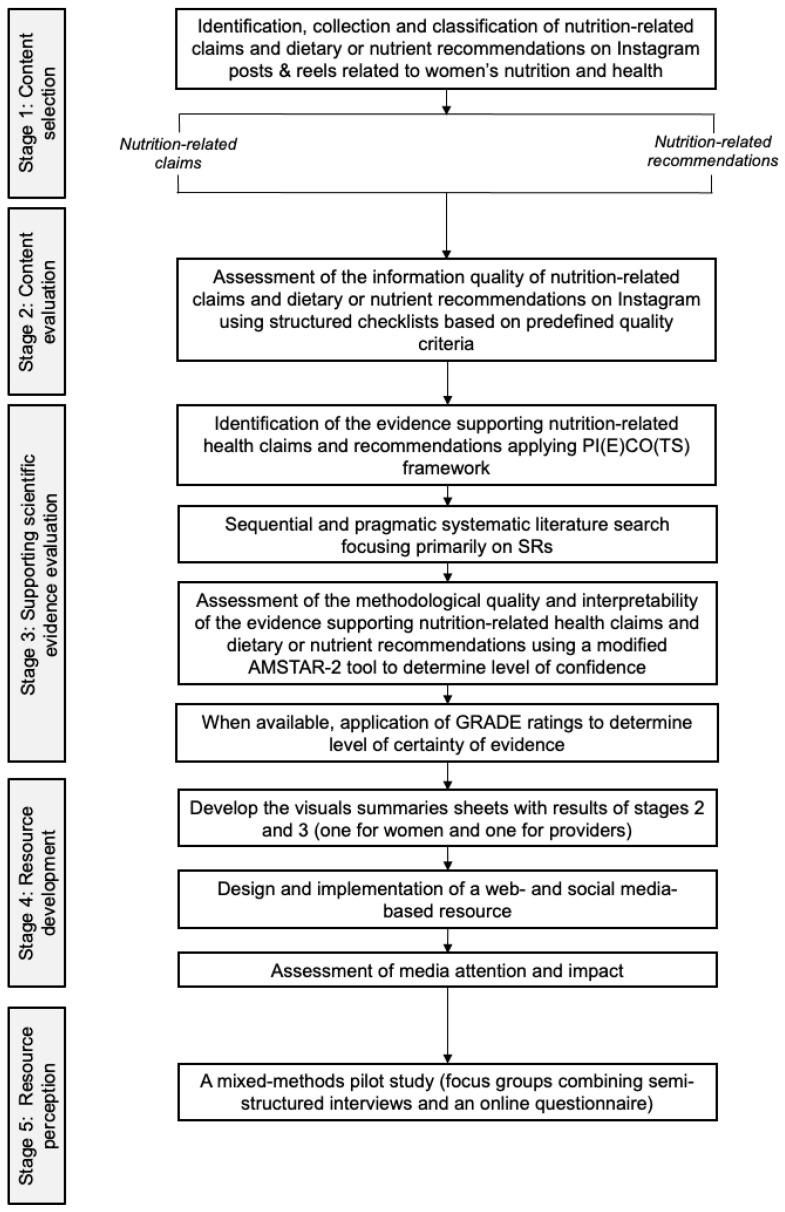
Overview of the NutriWomen project methodology. The study consists of five sequential stages: *(1)* Identification, collection, and classification of nutrition-related claims and recommendations on Instagram; *(2)* Assessment of the information quality of nutrition-related claims and dietary or nutrient recommendations; *(3)* Evaluation of the methodological quality and interpretability of the supporting scientific evidence; *(4)* Development of the NutriWomen digital platform for knowledge translation; and *(5)* Preliminary evaluation of the digital resource through a mixed-method pilot study exploring women’s perceptions and information needs across menopause stages.

The approach of the present project will follow a mixed-methods design, primarily based on retrospective content quality analysis of Instagram posts, followed by a secondary evaluation of the scientific confidence of the supporting evidence for nutrition-related claims and/or recommendations, using established methodologies [4,29,30,31]. The findings from this quantitative assessment (Stages 1–3) will directly inform the content and design specifications for the development of a digital resource for evidence-based nutrition communication (Stage 4). Additionally, a small-scale pilot study using focus groups will then be conducted to explore user perceptions and evaluate the usability of the developed platform (Stage 5), thus integrating both quantitative and qualitative insights to substantiate the project’s overall aims. Figure 1 summarizes the design of the project and each of its stages.

## 3. Detailed Procedure

**Stage 1: Identification, Collection, and Classification of Nutrition-Related Claims and Recommendations on Instagram**

Content analysis [32] will be performed on Instagram posts or reels published in Spanish and accompanied by text containing a claim related to the health effects of a specific diet, food, nutrient, or supplement [4] targeting women of reproductive age, women in perimenopause (45–55 years), menopause/postmenopause (50–60 years), or older women (60+ years) [33].

Inclusion criteria: (i) Nutrition claims in Spanish, Catalan, or English; (ii) Instagram publications consisting of an image or video with accompanying text; (iii) Messages in which the target population, intervention, or symptom/disease can be identified and contrasted with scientific evidence; and (iv) Publications created between 2023 and 2025.

Exclusion criteria: (i) Videos or images without accompanying text; (ii) Messages without any nutrition claim related to women’s health; (iii) Purely testimonial or opinion-based posts without verifiable claims; (iv) Publications lacking sufficient context for analysis or unverifiable content; (v) Advertisements or commercially oriented content; (vi) Videos less than 3 min; (vii) Posts from private accounts; and (viii) Duplicated content.

According to Instagram’s algorithm, “Top posts” represent the content that generates the highest engagement, considering factors such as posting time, user interactions, and alignment with Instagram’s recommendation guidelines. We will select the first 10 “Top posts” for each chosen hashtag to capture the most widely viewed and broadly disseminated content (i.e., #womenshealth, #femalehealth, #womenswellness). A list of hashtags related to dietary interventions and women’s health outcomes will be compiled based on the topics frequently addressed in current guidelines and systematic reviews to ensure scientific relevance. When Instagram content includes a direct recommendation to consume a specific food or supplement, this will also be coded to be compared with available guidelines and official sources (e.g., the European Food Safety Authority (EFSA) and the World Health Organization (WHO)). The content analysis will also incorporate trending or viral nutrition-related topics targeted at women of reproductive age or older, as identified by the research team through real-time monitoring of Instagram.

All searches will be conducted using a dedicated Instagram account created specifically for the project, ensuring that the “Top posts” associated with selected hashtags are based on post popularity while minimizing personalization bias in search results [29]. The coding process will follow several steps. First, a conducted pilot testing phase will be conducted in which one reviewer systematically codes an initial subset of posts and iteratively refines the codebook to improve clarity, consistency, and applicability of the categories. Second, to address the intra-rater reliability, the same reviewer will re-code a random subset of posts after a latency period, with discrepancies discussed to further refine operational definitions and decision rules. Third, to enhance external validity and reduce individual bias, a second researcher will independently review the final codebook and a sample of coded posts, resolving disagreements through discussion and consensus. When necessary, a third team member will be consulted to adjudicate any remaining uncertainties.

**Stage 2: Assessment of the Information Quality of Nutrition-Related Claims and Dietary or Nutrient Recommendations on Instagram**

First, one author will identify at least one post each month that meets the inclusion criteria and appears as a top post on Instagram. The engagement rate, calculated as (Likes+Comments)/(Number of Followers)×100, will be used to determine whether an Instagram post is worth analyzing.

The same author will then isolate claims regarding the nutrition intervention or outcome of interest for further analysis. Subsequently, the quality of nutrition-related information will be assessed using 14 predefined criteria, with a minimum score of 0 (poor quality) and a maximum score of 14 (high quality). This quality score was developed by combining a previously validated 10-item tool for assessing the quality of health websites with criteria specifically designed for social media content. The original items, created by Prados-Bo et al. and Ellis et al. [31,34] to evaluate the transparency, accuracy, and completeness of online health information, were adapted to the Instagram context and complemented with additional principles for health-related content on social media derived from Denniss et al. [30].

The resulting 14-item criteria to be used are: (1) States the name of the author(s) or contributor(s); (2) States credentials or affiliations of the author(s) or contributor(s); (3) States potential conflicts of interest; (4) Title is accurate and does not overpromise; (5) Language is understandable and avoids excessive technical terms; (6) Information is presented objectively and acknowledges nuances in supporting scientific publications; (7) Quantifies benefits in relative numbers; (8) Quantifies benefits in absolute numbers; (9) Reports potential harms; (10) Offers potential alternatives to the main intervention; (11) Includes a disclaimer to consult a healthcare provider; (12) Respects principles of privacy and confidentiality; (13) Images are visually appealing and reflect, rather than contradict, the information provided; and (14) Includes the link or full reference to scientific publications in indexed journals.

For the analysis of dietary recommendations on Instagram, it will also be recorded whether the content refers to a public health authority (e.g., EFSA, WHO). Each criterion will be rated as “satisfactory” or “unsatisfactory.”

One researcher will conduct the coding of information quality criteria, and all discrepancies will be resolved through discussion with a second researcher to ensure consistent final coding.

**Stage 3: Assessment of the Methodological Quality and Interpretability of the Evidence Supporting Nutrition-Related Health Claims and Recommendations**

To identify which nutrition-related claims and recommendations are supported by evidence, a structured question will be formulated using the PI(E)CO(TS) framework (Population or Problem, Intervention or Exposure, Comparison, Outcomes, Timeframe, Setting or Study Design). The PI(E)CO(TS) framework ensures clarity and specificity, facilitating a rapid and systematic evaluation [35,36].

Relevant health outcomes will be selected based on primary outcomes identified in systematic reviews (SRs). When available, standardized core outcome sets (COS) from initiatives such as the International Consortium for Health Outcomes Measurement (ICHOM, https://www.ichom.org) and Core Outcomes in Menopause (COMMA) will guide outcome selection. Similarly, COS from the COMET Initiative (https://www.comet-initiative.org) may inform the process. In the absence of specific COS, these frameworks will guide adaptation and prioritization of outcomes relevant to the evaluation context. Additionally, a systematic search in PubMed will be conducted, combining at least one MeSH term and free-text term with title restriction. Other stage-specific initiatives will also be explored to ensure comprehensive and standardized outcome selection. For each nutrition-related claim or recommendation, a maximum of three to four relevant outcomes will be selected following the GRADE framework [4,37].

To identify available scientific evidence, a sequential and pragmatic search will be conducted in PubMed and the Cochrane Database of Systematic Reviews. Combinations of MeSH and free-text terms, with title and/or abstract restrictions, will be used for each PI(E)CO(TS) component. In PubMed, appropriate filters (e.g., “Systematic Reviews” or “Guidelines”) will be applied. Evidence-based guidelines from official sources (e.g., EFSA, WHO, USDA, AESAN) as well as professional societies (e.g., ESPEN) will also be consulted when relevant.

SRs that use systematic methods in two or more databases and assess the certainty of evidence using the GRADE approach will be prioritized [4,31]. Briefly, GRADE is a widely recognized, transparent, and reproducible methodology adopted by the WHO and the Cochrane Collaboration to support clinical practice recommendations [24]. It classifies the certainty of evidence—reflecting confidence in the reliability of research results—into four levels: high (very confident in the results), moderate (moderately confident), low (limited confidence), or very low (very limited confidence), considering study design, risk of bias, consistency and precision of results, and directness of the evidence. SRs are selected because they synthesize and critically appraise available studies that address the PI(E)CO(TS) question and meet predefined inclusion criteria [35,36]. When GRADE-based SR are not available, non-GRADE SRs will be considered.

The methodological quality of included SRs will be assessed using a modified version of the Measurement Tool to Assess Systematic Reviews, version 2 (AMSTAR-2) was applied [23]. This version evaluates 18 critical items, including comprehensiveness of the search, study screening and data extraction processes, and risk of bias assessment. Additionally, it incorporates additional items to evaluate interpretability—specifically, the reporting of absolute effect estimates and the GRADE certainty of evidence. These interpretability items represent the key modification to AMSTAR-2 and were included to address critical criteria aligned with the project’s objectives, particularly regarding the certainty and clarity of reported outcomes [23].

For each outcome, the certainty of evidence within the same PI(E)CO(TS) question will be reported. Information on evidence type and GRADE domains will also be documented. Certainty ratings will be reported only when explicitly provided or when GRADE can be applied consistently. If certainty differs across critical or important outcomes, the lowest GRADE rating will be used as the overall certainty [4,31]. Finally, nutrition-related claims or recommendations will then be classified according to the supporting evidence, and their methodological quality and interpretability will be categorized using the modified AMSTAR-2 tool into high, moderate, low, or critically low confidence levels [38].

One researcher will code methodological quality and interpretability criteria, and any discrepancies will be resolved through discussion with a second researcher to ensure a unified final coding.

**Stage 4: Development of the Digital Resource: NutriWomen Platform**

The distinctive feature of this project is its approach to disseminating nutrition-related information to adult women by combining scientific rigor with accessible language. A dedicated website, the NutriWomen platform, will be developed to present the methodology, as well as the results of the quality assessment and scientific accuracy of the analyzed nutrition claims as they become available. The NutriWomen website will be built as a responsive, web-based platform using a modular architecture that separates the evidence-evaluation database from the presentation layer, which facilitates future updates and potential scaling to other languages or health domains. Second, we state that the site adheres to the Web Content Accessibility Guidelines (WCAG) 2.1 at the AA level wherever feasible (e.g., color contrast, alt text, keyboard navigation, plain-language infographics; see Figures S1 and S2 in the Supplementary Material), in line with current recommendations for health information websites. Third, the usability will be assessed through user-testing sessions (the focus group described in stage 5) and web analytics, using indicators such as task completion (e.g., ability to locate and understand a given claim evaluation), time on page, bounce rate, and qualitative feedback on clarity, relevance, and perceived trustworthiness. Finally, the content will be reviewed at least every six months by an editorial board (nutrition experts, clinicians, and digital-health specialists) following an internal updating protocol.

In parallel, Instagram will serve as the primary social media platform to drive traffic to the website and engage women.

For each Instagram nutrition claim or recommendation analyzed, we will develop a visual summary sheet in plain language (one tailored to women from the general public and another intended for health professionals) that will be disseminated through the project’s Instagram channel (Figures S1 and S2 in the Supplementary Material). Each evaluation will include the following sections, adapted in complexity and language according to the target audience [4]:

- Headline: States the question of the evaluation or the intervention and outcomes in one sentence.
- Quality of information: Provides the overall information quality grade and a summary of met and unmet quality criteria (see “Stage 2” for further details).
- Quality and certainty of the evidence: Provides the overall score of the methodological quality of the systematic review and a summary of met and unmet quality criteria (see “Stage 3” for further details), whenever there is a SR that evaluates the claim of interest. This section will only be included in the version intended for healthcare providers, while the general public version will feature a simplified visual summary.
- Critical thinking advice: Includes a brief reflection or tip designed to assist the audience in identifying inaccurate claims or recommendations and making informed decisions regarding health and nutrition.

The NutriWomen platform (https://web.ub.edu/ca/web/projecte-recerca-nutridones) will be responsive, multi-layered, and available in Spanish, Catalan, and English. It will be hosted by the University of Barcelona, which will provide technical support for its development and maintenance.

Online attention and impact will be assessed using Altmetric. Website performance metrics (number of visits, visits per user, and top engaging countries) will be collected via Google Analytics. Collaboration metrics with key stakeholders, such as partnerships with influencers, women’s associations, community centers, and professional bodies, will also be documented. The number of newsletter subscribers will also be collected as a metric of social relevance. Promotion of the hashtags #NutritionForWomen #NutricionParaMujeres for Spanish speakers and #NutricióPerDones for Catalan speakers in each publication will help the NutriWomen team monitor and track social-media engagement (profile visits, followers, comments, shares, reach and impressions). The project website will also include a contact form to gather user feedback, which will inform future analyses of information quality and scientific evidence based on users’ needs.

**Stage 5: Preliminary Evaluation of the Digital Resource through a Mixed-Methods Observational Study Exploring Women’s Perceptions, Information Needs, and Preferences**

For the preliminary evaluation phase, the menopausal transition will be prioritized because mid-life represents a particularly vulnerable period for the onset of functional limitations and the early stages of disability [39]. Following the WHO empowerment model, menopause should be understood as more than just the treatment of specific symptoms. It is essential to develop and disseminate information that presents menopause as a normal, healthy stage in women’s lives. Across various health domains, there is growing evidence that empowerment is an effective tool for enhancing self-management of health, which can also contribute to creating menopause-friendly environments at work and at home, and to addressing persistent stigma and gender-based ageism. However, to be empowered, women must be properly informed and listened to [40,41,42]. Thus, it is essential to develop and disseminate information that presents menopause as a normal, healthy stage in women’s lives. Across various health domains, there is growing evidence that empowerment is an effective tool for enhancing self-management of health, which can also contribute to creating menopause-friendly environments at work and at home, and to addressing persistent stigma and gender-based ageism. In this context, we propose a preliminary mixed-methods observational study designed to assess women’s information needs, preferences, and perceptions regarding menopause, a critical life stage in which many women report a strong interest in receiving evidence-based nutrition information [7], as well as to collect feedback on the usability and relevance of the NutriWomen digital platform [43]. The study will adopt a multiphase sequential design incorporating both exploratory and explanatory phases to integrate qualitative and quantitative evidence.

### 3.1. Participants

This study will be conducted with women at different stages of menopause (perimenopausal, menopausal and postmenopausal) to refine the digital resource and ensure that it is accessible, understandable, and relevant to the target audience before formal validation. Participants (n = 42) will be recruited through bulletin boards in local city councils, health centers and private clinics in the Barcelona and Tarragona areas. Recruitment will also take place via social media, including Facebook women’s association groups and influencers (10,000 followers) specializing in women’s health on Instagram. Each potential participant will complete an online screening questionnaire to verify eligibility (e.g., menopausal status, age range, and ability to provide informed consent) and will then be invited to take part in a focus group interview.

### 3.2. Data Collection and Analysis

The study will follow a sequential exploratory design beginning with three focus group interviews according to menopausal stage (n = 14 women each), inspired by previous research conducted in Sweden [44]. A semi-structured interview (SSI) will explore: Women’s experiences of menopause; Attitudes and knowledge about menopause; Experiences with menopausal care; Sources of information used; Information needs and preferences for future menopausal information [41]. Each focus group will be moderated by a researcher trained in qualitative methods, with another researcher observing and taking notes. Sessions will be audio-recorded and transcribed verbatim. After the discussion, participants will be introduced to the NutriWomen platform, and feedback will be gathered to evaluate accessibility, comprehensibility, and perceived usefulness. The qualitative analysis of the focus groups will follow a four-step process using systematic text condensation, inspired by Malterud [45]. In the first step, the transcripts will be read several times to gain an overall understanding of the findings; In the second step, broad preliminary themes will be identified, and meaning units related to these themes will be extracted; In the third step, the meaning units will be condensed while preserving their core essence, and sections with similar content will be defined and coded; In the fourth and final step, the codes will be grouped into subcategories, which will then be organized into higher-level categories reflecting more abstract concepts.

## 4. Expected Results

The integrated approach combines content analysis of selected Instagram posts, an assessment of the evidence behind nutrition-related claims, and a mixed-methods focus group to identify information needs and collect feedback on the NutriWomen platform. Together, these components are intended to generate rigorous and transferable insights for both women and healthcare professionals and to guide the iterative refinement of this platform.

To the best of our knowledge, NutriWomen is among the first initiatives that, through the application of a systematic methodology, evaluates nutrition claims circulating on social media using a research-based methodology and aim to translate the results into accessible, evidence-based information specifically tailored to women’s health needs. The NutriWomen platform is designed to support women in managing their health across the life course, from the reproductive years to menopause and frailty, by offering clear, unbiased, and evidence-based nutrition information that may help inform health decisions and foster personalized self-care. The platform will include decision-support tools (e.g., infographics and visual summaries) to facilitate comprehension and enable the personalization of dietary strategies for managing, for example, menopausal vasomotor symptoms, genitourinary infections, and other gender-specific health conditions.

From the mixed-methods evaluation, we anticipate that quantitative results will help describe patterns in health and nutrition literacy across menopausal stages, while qualitative findings will provide in-depth insights into women’s experiences, perceived barriers to reliable information, attitudes toward self-management, and feedback on usability, clarity, and relevance of the platform.

Overall, the results are expected primarily to guide the iterative refinement of NutriWomen, improving accessibility, clarity and alignment with users’ preferences, and to inform the development of targeted, evidence-based educational materials that may support more informed health decisions. Based on prior work showing that decision aids could improve patient’s understanding and support shared decision-making, NutriWomen may yield similar benefits; however, any improvement in health or nutrition literacy, behavior or clinical outcomes will need to be confirmed by empirical evaluation.

Nevertheless, these tools are not intended to replace individual clinical assessment or professional dietary counseling. NutriWomen was created in response to the need to actively counteract widespread nutrition misinformation, particularly on Instagram, and aims to position itself as a reliable online source of guidance. However, the scope of its impact will depend on women’s awareness of the platform, sustained engagement over time, and the NutriWomen team’s ability to create content that is both appealing and useful.

Future longitudinal studies will be required to determine the longer-term impact of the platform and its applicability across diverse cultural and socioeconomic settings. At this stage, NutriWomen will focus on questions for which at least one SR exists, while topics without an SR will be reported as evidence gaps, with the possibility of incorporating high-quality primary studies in future iterations as the project evolves. Dissemination will occur through peer-reviewed publications, scientific meetings and the NutriWomen website and social media channels.

### 4.1. Ethical Considerations and Dissemination

This work will be carried out in accordance with The Code of Ethics of the World Medical Association (Declaration of Helsinki). Women who agree to participate in Stage 5 of the study will be provided with both oral and written information about the project. They will be informed that participation is voluntary, that they may withdraw from the study at any time without providing a reason and without facing any negative consequences, and that no individual will be identifiable in the results. Written informed consent will be obtained prior to participation.

The study will be approved by the Ethics Committee designated by the Bioethics Commission of the University of Barcelona (CBUB). Results will be disseminated through a dedicated website (https://web.ub.edu/ca/web/projecte-recerca-nutridones), blog, social media channels, academic publications, and local events. The study protocol will be publicly available to promote transparency, reproducibility, and adherence to FAIR (Findable, Accessible, Interoperable, and Reusable) principles. Focus group participants (but not questionnaire respondents) will be compensated with a small e-book on nutrition during menopause, in recognition of their time. This aligns with NutriWomen’s mission to enhance women’s health literacy by creating tailored educational resources that empower them to address menopausal symptoms and chronic conditions, thereby supporting optimal health management.

### 4.2. Public Involvement

Public involvement is central to the design and implementation of this web-based resource, which adopts a participatory science approach. A focus group composed of 42 women at different stages of menopause (perimenopausal, menopausal, and postmenopausal) will be conducted to ensure that their perspectives directly inform both the development and evaluation of the website.

Participants will be invited to provide feedback on the clarity, relevance, and accessibility of the content, as well as to share their informational needs and preferences related to menopause. Their input will guide the refinement of both the resource and the communication strategy, ensuring alignment with real user expectations and preferences.

Additionally, focus group participants will be asked to comment on the usability of the tool and contribute to the interpretation of results. This participatory approach aims to enhance the relevance, acceptability, and overall impact of the final website.

## Figures and Tables

**Table 1:** Definition of terms.

Term	Definition ^1^
Nutrient recommendations (or dietary reference intakes) [26]	Recommendations that define the daily intake levels of essential nutrients (expressed in units such as mg or g per day) that are considered adequate to meet the physiological needs of almost all healthy individuals in a population.
Dietary recommendations (or dietary guidelines, also known as food-based dietary guidelines [27]	Science-based guidelines that provide advice for populations on healthy eating patterns. Rather than focusing on individual nutrients, dietary recommendations guide food decisions to promote overall health and prevent chronic diseases. They are designed to support informed decisions about diet and lifestyle at the population level.
Claim [28]	Our use of the term ‘nutrition claims’ is not related to food product labeling or marketing but rather to general health-related statements or assertions that can impact women’s health.

^1^ Definitions are provided to ensure consistency in terminology when discussing the methodology used.

## Data Availability

The original contributions presented in this study are included in the article and Supplementary Materials. Further inquiries can be directed to the corresponding author.

## Supplementary Materials

The following supporting information can be downloaded at: https://www.mdpi.com/article/10.3390/nu18010020/s1, Figure S1: Example of an evidence-informed visual summary adapted for women from the public; Figure S2: Example of an evidence-informed visual summary adapted for healthcare professionals. References [46,47,48,49,50] are cited in Supplementary Materials.

## Abbreviations

The following abbreviations are used in this manuscript:

AESAN	Agencia Española de Seguridad Alimentaria y Nutrición
AMSTAR-2	A MeaSurement Tool to Assess Systematic Reviews, version 2
CBUB	Comité de Ética designado por la Comisión de Bioética de la Universidad de Barcelona
COMET	Core Outcome Measures in Effectiveness Trials Initiative
COMMA	Core Outcomes in Menopause
COS	Core Outcome Set
EFSA	European Food Safety Authority
ESPEN	European Society for Clinical Nutrition and Metabolism
FAIR	Findable, Accessible, Interoperable, and Reusable
GRADE	Grading of Recommendations Assessment, Development and Evaluation
ICHOM	International Consortium for Health Outcomes Measurement
MeSH	Medical Subject Headings
PI(E)CO(TS)	Population or Problem, Intervention or Exposure, Comparison, Outcomes, Timeframe, Setting or Study Design
SR	Systematic Review
SSI	Semi-structured interview
USDA	United States Department of Agriculture
WHO	World Health Organization
