# Supplementary figures and images for: NutriWomen, Novel Evidence-Based Web Platform to Support Women’s Health, Nutrition Decisions and Address Nutrition Misinformation on Social Media: Protocol for a Digital Tool Development
Source: Nutrients. 2025 Dec 19;18(1):20. doi: 10.3390/nu18010020 (PMC12787998; doi:10.3390/nu18010020)

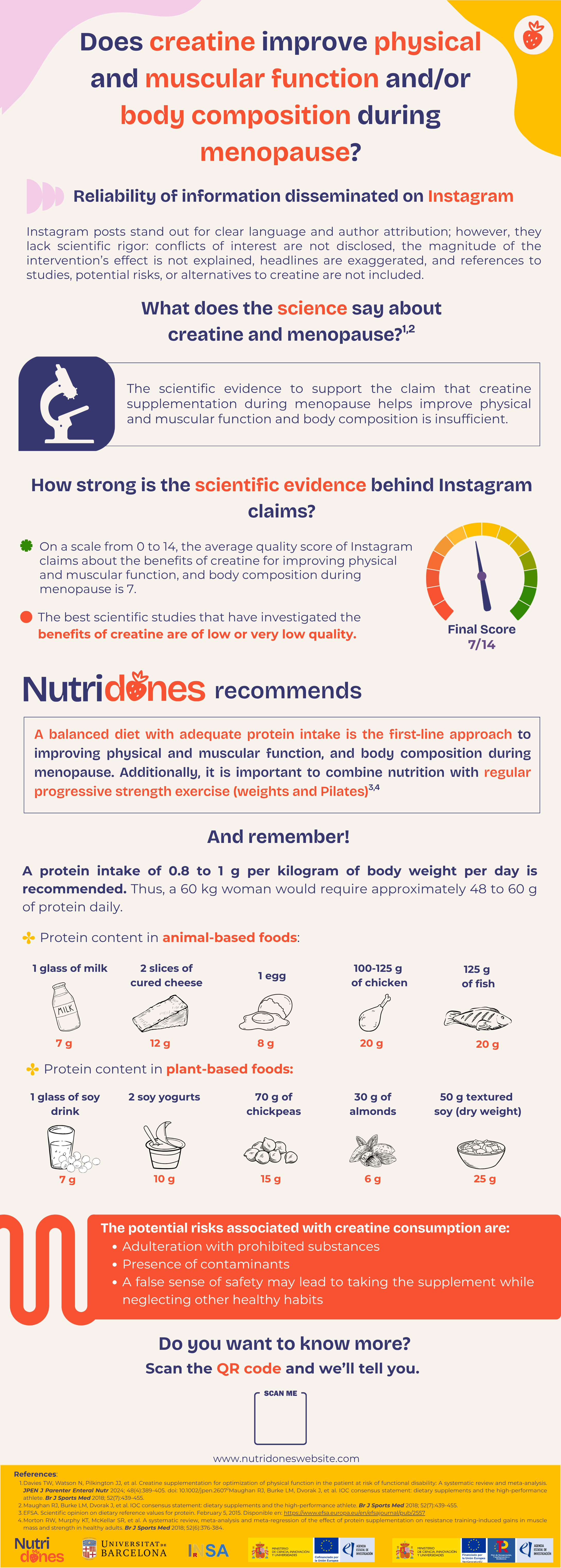

Supplement: Supplementary file 1 [file nutrients-18-00020-s001.zip › Figure S1_Example of an evidence-informed visual summary adapted for women from the public.png]

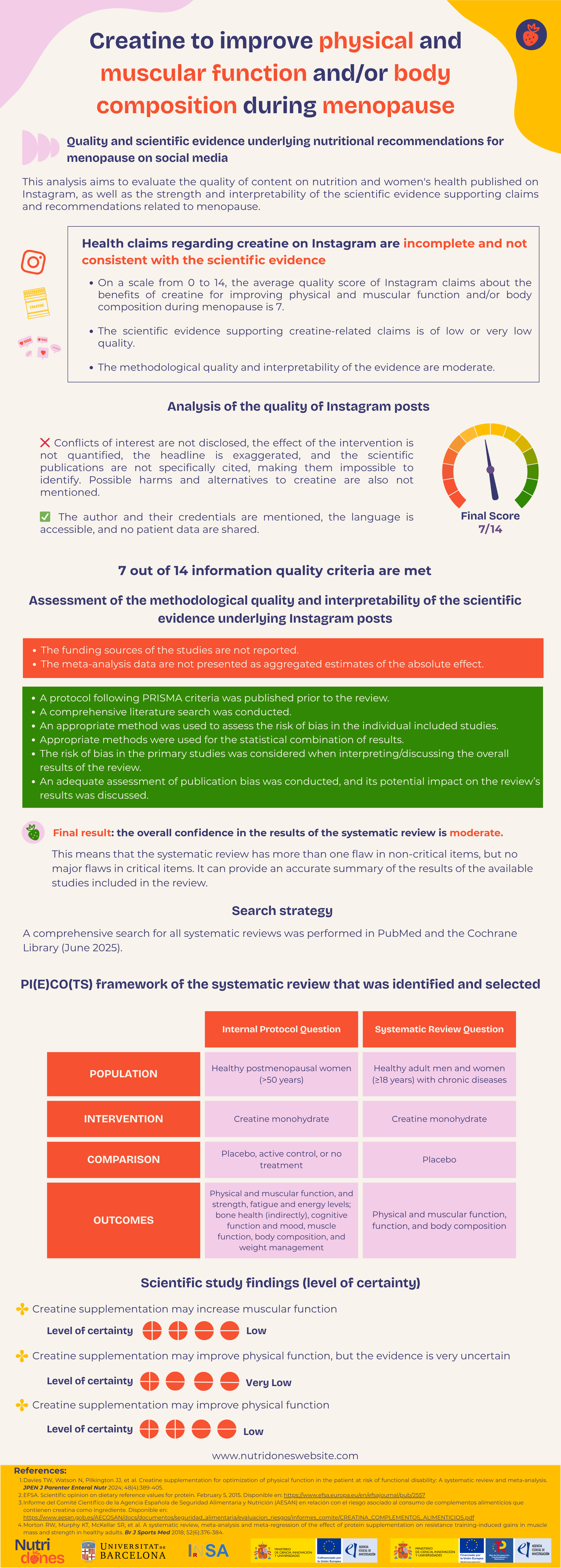

Supplement: Supplementary file 1 [file nutrients-18-00020-s001.zip › Figure S2_Example of an evidence-informed visual summary adapted for healthcare professionals.png]
